# Navigating the Nexus of Food Insecurity, Anxiety, and Depression in the Face of Climate Change: A Longitudinal Study in Rural Kenya

**DOI:** 10.1155/da/5510493

**Published:** 2025-11-07

**Authors:** Michael Goodman, Lauren Raimer-Goodman, Heidi M. Hagen McPherson, Dawit Woldu, Shreela Sharma, Ryan Ramphul, Fridah Mukiri, Agnes Maigallo

**Affiliations:** ^1^Department of Population Health and Health Disparities in the School of Public and Population Health, University of Texas Medical Branch, Galveston, Texas, USA; ^2^Department of Pediatrics, University of Texas Medical Branch, Galveston, Texas, USA; ^3^Center for Health Equity, The University of Texas Health Science Center, Houston School of Public Health, Houston, Texas, USA; ^4^Department of Anthropology, University of Houston-Clear Lake, Clear Lake, Texas, USA; ^5^Department of Epidemiology, University of Texas School of Public Health, Houston, Texas, USA; ^6^Sodzo International, Maua, Meru County, Kenya; ^7^Chuka University, Chuka, Tharaka Nithi County, Kenya

## Abstract

**Objective:**

This study aims to address critical gaps in understanding the bidirectional relationships between food insecurity, anxiety, and depression in Meru County, Kenya. By employing a cross-lagged panel analysis, we seek to clarify these temporal dynamics, contributing to the design of targeted interventions that integrate food security and mental health in the context of climate change.

**Methods:**

A cross-lagged panel analysis was conducted using data from 362 adult participants in a community-based empowerment program (2023) in Meru County, Kenya. Participants completed self-report measures of food insecurity, anxiety, and depression at two time points, 11 weeks apart.

**Results:**

Food insecurity (T1) predicted subsequent anxiety and depression (T2), controlling for within-variable, within-time, and control-variable correlations. Village-level food insecurity (T1) was correlated with significantly higher anxiety (T2). Additionally, anxiety (T1) predicted higher subsequent food insecurity (T2).

**Conclusion:**

Food insecurity and anxiety have a complex bidirectional relationship. Interventions that address food security, mental health, and the psychosocial factors that promote adaptation to food-insecure environments are essential for promoting the well-being of individuals and communities in the face of climate change.

## 1. Introduction

Climate change presents an increasingly urgent threat to human well-being through multiple pathways. One such pathway includes linkages between climate change and food production within smallholder farming societies such as within Kenya and across sub-Saharan Africa [1, 2]. Through disruption to rainfall patterns, degrading soil quality, and high temperatures [3–5], crop yields have declined and are project to continue declining within societies whose main food source is smallholder farms [6, 7]. Any effort to determine or project the toll of climate change to human health must be informed by mechanisms through which food security—the state in which “all people at all times have both physical and economic access to sufficient food to meet their dietary needs for a productive and healthy life” [8, 9]. Food insecurity indicates an inability to achieve these conditions through socially acceptable means [10, 11].

In addition its consequences to human development [12], food insecurity has increasingly been linked to mental health challenges [13]. However, most current research assessing food insecurity and mental health depends on cross-sectional data from high-income countries; for example, the meta-analysis reported by Pourmotabbed et al. [14] relied on data from seven high-income countries, two upper–middle-income countries, and one middle-income country. Further, the Pourmotabbed et al. [14] meta-analysis found only one longitudinal study comparing relationships between food insecurity and mental health [15]. Pourmotabbed et al. [14] report consistent associations between food insecurity and depression and stress, but inconsistent findings between food insecurity and anxiety. Cross-sectional survey data from 149 countries demonstrate associations consistent with dose–response relationships between food insecurity and worry—a central feature of anxiety [16], though this study was not included in the Pourmotabbed et al. [14] review, presumably due to measurement differences. Limited assessment of food insecurity and mental health in low-income countries represents a knowledge gap because these regions face disproportionate burdens of food insecurity and inadequate mental health resources [17]. Most existing research is derived from high-income settings, which may not account for the sociocultural and economic contexts that influence these relationships in low-income countries [14].

Depression and anxiety, two common mental health disorders, are characterized by distinct features: depression involves persistent sadness, loss of interest or pleasure, and functional impairment, whereas anxiety is marked by excessive worry, fear, and physical symptoms like restlessness and fatigue [18, 19]. Both disorders can undermine cognitive and emotional functioning, reducing individuals' capacity to engage in income-generating activities, maintain employment, or manage household resources, thereby exacerbating food insecurity [13]. Conversely, mental assets, such as resilience and adaptability, may buffer against the adverse effects of food insecurity and promote better outcomes [20, 21].

Understanding the relationships between food insecurity and mental health in low-income and rural settings like Meru County, Kenya, requires attention to both individual and community-level dynamics. In collectivist societies, shared environmental conditions, communal resource management, and social cohesion significantly influence food security and mental well-being, making village-level analysis a meaningful approach. Aggregating findings at the village level allows for the identification of patterns that reflect the socioecological contexts within which food insecurity and mental health outcomes are embedded, offering insights into potential collective interventions.

### 1.1. Study Aims

This study assesses cross-lagged panel relationships of food insecurity, depression, and anxiety among adult participants (*n* = 362) in a community-based empowerment program in Meru County, Kenya. Specifically, it aims to determine whether these relationships are significant at the individual level and whether correlational patterns are observed at the village-aggregated level. By examining these dynamics, the study contributes to understanding both individual and community-level factors influencing mental health and food insecurity in resource-constrained contexts.

## 2. Methods

### 2.1. Geographical Location

**Figure 1:**
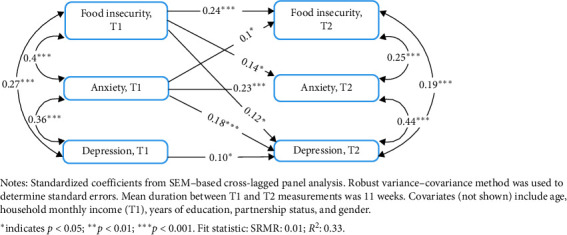
Cross-lagged panel model of food insecurity, anxiety, and depression among adults in rural Kenya (*n* = 362). Model estimated using structural equation modeling with robust standard errors, controlling for age, income, education, gender, and partnership status. Arrows indicate significant temporal pathways (T1 → T2). Coefficients represent standardized path estimates; *p*  < 0.05.

This study was conducted in Central and South Igembe subcounties of Meru County, Kenya, which is located just north of Mount Kenya. Food security during the study period are shown in Figure 1a,b. At the start of the study period, acute food insecurity was considered stressed in the study locations, but had progressed to “crisis” levels according to Famine Early Warning Systems Network [22].

### 2.2. Study Setting

This study used data from participants in a community-based empowerment program that has previously been shown to improve mental health, parenting behaviors, income, and reintegration outcomes for street-involved children and youth [23–25]. The program involves local recruitment, uses village-level group structures, and incorporates culturally relevant practices such as microfinance and community leadership development, which are driven by local participants rather than external entities. The intervention combines group-based microfinance practices, leadership development, and a novel behavioral health curriculum to promote values-based growth [26]. A previously developed index of lending group-affiliated interpersonal trust, norms of reciprocity, sense of belonging, and cohesion was shown to be cross-sectionally associated with lower food and water insecurity [27]. The aspirational goal of the intervention, guided by our Flourishing Community Model [26], is to foster communities that are supportive, resilient, generative, compassionate, curious, responsive, and self-determining. Survey-based questionnaire data were collected in February 2023 (T1) and May 2023 (T2). The mean (sd) amount of time between observations was 11.5 (4) weeks.

### 2.3. Recruitment Practices

Program participants were recruited through a combination of active and passive recruitment. Active recruitment began at the village level, where families with children living on the streets or families engaged in HIV care at Ministry of Health clinics were identified [26]. These index families then recruited 25–29 other families to join a microfinance group. Additional families joined the program through passive recruitment, mostly by word of mouth [23]. Participants were, therefore, nested within groups, which were nested within villages. Ten villages were represented in the current study. As the program focuses on supporting caregivers—the overwhelming majority of whom are women and consistent with the composition of Village Savings and Lending Associations globally, the majority of participants (~90%) are women [28, 29].

### 2.4. Participant Selection

Once groups were formed, program evaluation was conducted to track changes in key variables over the duration of active program participation. Participants in this study were randomly selected during routine group microfinance activities using a random number generator. All willing participants (97.8% of all participants) in the first six groups formed within a village were invited to draw a folded piece of paper from an opaque bag, with “1” indicating selection and “0” indicating non-selection – from each group of 30 participants, 7 were selected for the study. Each established group within a village was invited to participate in the evaluation. There were 362 individual participants selected from 60 groups across 10 villages with paired T1 and T2 data in this study. Sample size was driven by limited financial resources to secure cohort-data and inform a future cluster-randomized control trial to improve mental health and food security outcomes.

### 2.5. Study Design

This study used an interventional cohort design, with two data collection waves.

### 2.6. Measures

The survey questionnaire was developed in English, translated into the local language (Kimeru), and then, back-translated into English to ensure accuracy and consistency. The survey was administered by a team of trained local experts from two Kenyan universities in the study county.

### 2.7. Primary Measures

This study examines the cross-lagged relationships between three primary variables: generalized anxiety, depression, and food insecurity. Responses to all primary variables are continuous summations of item responses. Mean imputation was used for missing items in all scales where the number of missing items was fewer than 10% of the total scale; there were no respondents with more than 10% missing items on included scales.

### 2.8. Generalized Anxiety

Generalized anxiety was measured using the GAD-7, a 7-item measure that assesses how often respondents have experienced symptoms of generalized anxiety in the past 2 weeks. Generalized anxiety is a common mental health disorder marked by persistent and potentially debilitating worry, sustaining negative affect and contributing to dysfunctional psychobehavioral coping [30]. The response scale is 4-point Likert-type, ranging from “not at all” to “nearly every day.” The GAD-7 has been validated in Kenya and other global populations [31]. The GAD-7 had excellent reliability in the present sample (*α* = 0.87 at T1; *α* = 0.91 at T2).

### 2.9. Depression

Depression was measured using the 21-item Beck's Depression Inventory-II (BDI-II; [32]). The BDI-II measures symptoms such as sadness, hopelessness, guilt, irritability, and difficulty concentrating over a 2-week recall period. Each item is rated on a 4-point scale, and the total score is calculated by summing the ratings for all 21 items. A higher score on the BDI-II indicates more severe depressive symptoms. The BDI-II showed acceptable reliability in both time points (*α* = 0.82 in T1; *α* = 0.83 at T2). The BDI-II has been used in various cultures, including within Kenya, demonstrating strong predictive and discriminant validity [33–37].

### 2.10. Food Insecurity

Food insecurity was measured using the Household Food Insecurity Access Scale (HFIAS; [38]), a nine-item scale that assesses the severity of food insecurity in households over the past 4 weeks. The HFIAS is one of the most widely used and well-validated measures of food insecurity in the world [39]. Each item on the HFIAS is rated on a 4-point scale, and the total score is calculated by summing the ratings for all nine items. A higher score on the HFIAS indicates more severe food insecurity. The HFIAS showed excellent internal reliability at T1 (α = 0.94) and T2 (α = 0.95).

### 2.11. Control Measures

Control measures were treated as time-invariant, measured at T1. Control measures included age, estimated household monthly income, years of education, marital status, and gender. Age, income, and years of education were measured as continuous variables. Marital status (married/cohabitating vs. not married/not cohabiting) and gender (man or woman) were included as binary variables. All control variables have been previously found to correlate with depression, anxiety, and food insecurity [40–44].

### 2.12. Analytical Approach

All scale variables were calculated by averaging item responses.

Descriptive data analyses include univariate descriptions of primary measures between T1 and T2. We used Wilcoxon signed-rank tests to evaluate equivalence of primary variables between T1 and T2. In bivariate analysis, Spearman rank correlation coefficients were calculated using Bonferroni-adjusted *p*-values to compare all primary and control measures.

At the individual-level, we conducted a cross-lagged panel analysis using structural equation modeling (SEM) with robust standard errors to account for nonnormal or clustered data [45]. The preliminary model considered all pathways between T1 and T2 primary variables and included all control variables in all pathways. Following a backward stepwise model-building approach, we recalculated the model until all variable pathways had significance tests *p*  < 0.20 and considered statistical significance at *α* < 0.05. Figure 1 depicts this SEM-enabled cross-lagged panel model.

**Figure 2:**
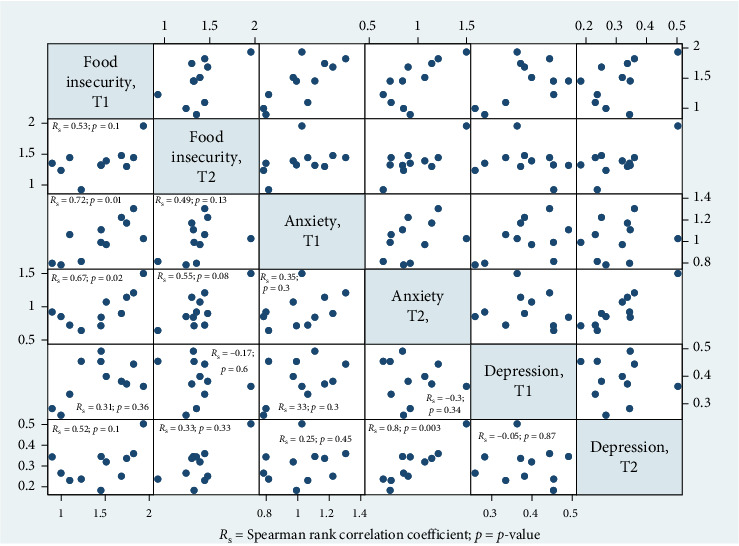
Village-level correlations between food insecurity, anxiety, and depression in rural Kenya (*n* = 10 villages). Spearman rank correlation coefficients with Bonferroni-adjusted *p*-values are shown for average values within villages at T1 and T2. Significant correlations (*p*  < 0.05) are highlighted.

We employ a cross-lagged panel analysis to examine the bidirectional correlations between food insecurity and mental health (e.g., depression and anxiety) overtime. This statistical technique permits assessment of one variable at an earlier time point (e.g., food insecurity) predicts changes in another variable (e.g., mental health) at a later time point and vice versa. By controlling correlations between variables at both time points and correlations within variables between time points, the approach helps to clarify whether food insecurity predicts future changes in mental health, mental health predicts future food insecurity, or whether these relationships are reciprocal. At the village-level, we calculated weighted average responses within each village to explore Spearman correlation analysis of primary variables.  Figure 2 depicts the resultant correlation matrix, Spearman rank correlation coefficient, and respective *p*-value for the 10 included villages. We provide the average (sd) values for all villages in Table S1.

All statistical analyses were conducted using STATA v.18.1 [46].

### 2.13. Ethical Considerations

All data were collected following ethical approval from the institutional review boards at the Kenya Methodist University (KeMU/SERC/21/2020) and the University of Texas Medical Branch (19-0241). The research was conducted under a research permit provided by the Kenyan National Commission for Science, Technology and Innovation (NACOSTI). All participants provided informed verbal consent prior to engaging in the interviews. Following the preferred approach to compensation indicated by participants in interventional program, internal lending groups of each participant received $1 in exchange for their participation in the study.

## 3. Results

Table 1 shows the univariate analyses of variables and the probabilities that primary variables have equivalent values at T1 and T2. Food insecurity, anxiety, and depression all significantly declined between T1 and T2 (*p*  < 0.01 for each comparison). The average household monthly income was $35 at T1. The mean (sd) age was 41.7 (12.6) years. The mean (sd) years of formal completed school was 5.2 (3.2). The percentage of respondents who reported being married or living with a partner as though married was 76%, and 92% of respondents were women.

Table 2 shows the Spearman rank correlation coefficients with Bonferroni-adjusted *p*-values. Food insecurity (T1) was significantly correlated with all variables, except age. Food security (T2) was significantly correlated with anxiety (T1 and T2), depression (T2), and education (inversely, T1). Anxiety (T1) was significantly correlated with anxiety (T2) and depression (T1 and T2). Anxiety (T2) was significantly correlated with depression (T2). Depression (T1) was significantly correlated with depression (T2). Years of formal schooling were significantly correlated with higher monthly income and lower age (T1).

Figure 1 shows the model calculated from using SEM with robust standard errors to calculate a cross-lagged panel analysis, controlling for age, income, gender, education, and partnership status. Food insecurity (T1) predicted increased depression (T2; *r* = 0.12, *p*  < 0.05), anxiety (T2; *r* = 0.14, *p*  < 0.05), and food insecurity (T2; *r* = 0.24, *p*  < 0.001). Anxiety (T1) predicted higher food insecurity (T2; *r* = 0.1, *p*  < 0.05), anxiety (T2; *r* = 0.23, *p*  < 0.001), and depression (T2; *r* = 0.18, *p*  < 0.001). Depression (T1) predicted increased depression (T2; *r* = 0.1, *p*  < 0.05).

Figure 2 shows the correlation matrix, respective Spearman rank correlation coefficients and *p*-values between primary variables averaged at the village level. As shown, food insecurity (T1) was significantly correlated with anxiety (T1; *r* = 0.72, *p*=0.01; T2; *r* = 0.67, *p*=0.02). Anxiety (T2) was significantly correlated with depression (T2; *r* = 0.8, *p*=0.003).

## 4. Discussion

To support research and response to impacts of climate change on mental health, we analyzed temporal relationships between food insecurity, anxiety, and depression at individual- and village-levels. We found food security predicts subsequent anxiety and depression, controlling for within-variable, within-time, and control variable correlations at the individual-level. At the village-level, we found that aggregated food insecurity (T1) was correlated with significantly higher anxiety (T2). These findings indicate that food insecurity may influence the etiology of anxiety at multiple socioecological levels and depression at the individual-level.

Not only did food insecurity predict higher subsequent depression and anxiety, but also anxiety (T1) predicted higher subsequent food insecurity. As within-time (T2) correlations between food insecurity and anxiety should account for general experiences of worry captured by the food insecurity measure, more research is required to understand psychological mechanisms promoting adaptation to food-insecure environments. As common mental disorders, including anxiety, have previously been shown to undermine productivity in addition to adding direct costs to healthcare expenditures [47, 48]. We know of no study that explores how psychological mechanisms, including anxiety, may influence adaptation to food insecure conditions. Understanding and supporting the behavioral, mental, social, and policy conditions that enable positive adaptation to climate change are desperately needed.

That village-level food insecurity was significantly correlated with subsequent anxiety indicates that rural locations where food systems are more adversely affected by climate change may experience subsequently higher levels of anxiety [49, 50]. Mental health promotion efforts should explore the use of existing secondary data on food security, rainfall disruption, and other indicators impacted by climate change to promote mental health across whole villages. The potential for village-level intervention on food security and mental health is indicated by the present study findings. The observed associations, though statistically significant, were modest in magnitude, suggesting that while food insecurity may influence mental health, interventions should consider a range of other socioeconomic and psychological factors to comprehensively improve mental health [16].

As food insecurity, anxiety, and depression all significantly improved after 11 weeks in the program, further evaluation of the interventional setting—particularly through a cluster-randomized control trial—is necessary. While the timing between T1 and T2 was relatively short (11 weeks), this period saw food insecurity intensify (Figure S1a,b). In contrast, food insecurity, depression, and anxiety all significantly decreased among study respondents in these locations. Whether and how the intervention supports these improvements should be explored in a mechanistic study with robust qualitative data to nuance findings.

Our previous research found that water insecurity, but not food insecurity, was temporally correlated with anxiety [27]. In the current study, food insecurity was linked to anxiety, and a secondary analysis using a shorter, 2-item version of the food insecurity scale supported these findings. This contrasts with earlier results that did not identify a link between food insecurity and anxiety, suggesting that the relationship is influenced by factors beyond the choice of measurement scale. These findings align with prior research, highlighting the need for further exploration of contextual factors, such as how food is secured and the psychological assets required to adapt to food-insecure environments. Such dynamics may vary significantly across high- and low-income contexts [14]. Understanding these differences is critical for identifying why food insecurity and anxiety are linked in some settings, but not others.

### 4.1. Limitations

The present study has several limitations that should be considered when interpreting the findings. First, the study relied on self-report data, which is susceptible to bias. Individuals may underreport or overreport symptoms of anxiety, depression, and food insecurity due to social desirability or other factors. Additionally, the use of interviewer-administered questionnaires may have introduced interviewer bias, as interviewers may have inadvertently influenced participants' responses.

Second, the study was conducted in a specific community in Kenya, and the findings may not be generalizable to other populations or settings. Cultural factors and other contextual factors may influence the relationships between food insecurity, anxiety, and depression. Further research is needed to examine these relationships in different populations and settings.

Third, the short follow-up period (11 weeks) limits the ability to assess long-term trends and causality in the relationships between food insecurity and mental health. Additional data points over a longer timeframe could provide a more comprehensive understanding of these dynamics.

Fourth, the study did not assess potential mediators or moderators of the relationships between food insecurity, anxiety, and depression. Future research should investigate potential factors that may influence these relationships, such as social support, coping mechanisms, and access to mental health services.

Despite these limitations, the present study provides important insights into the complex relationships between food insecurity, anxiety, and depression. The findings underscore the need for holistic interventions that address food security, mental health, and the psychological factors that promote adaptation to food-insecure environments.

## 5. Conclusions

This study investigated the temporal relationships between food insecurity, anxiety, and depression among adult participants in a community-based empowerment program in Meru County, Kenya. Our findings indicate a complex interplay between these variables at both the individual and village levels. Food insecurity was found to predict subsequent anxiety and depression at the individual level, while village-level food insecurity was correlated with higher anxiety. Additionally, anxiety predicted subsequent food insecurity, suggesting a bidirectional relationship between these variables.

These findings highlight the importance of community-level support and community resilience in promoting mental health in the face of food insecurity. Village-level associations between food insecurity and anxiety suggest that communities with stronger social networks and collective coping mechanisms may be better able to buffer the negative impacts of food insecurity on mental health. Further research is needed to understand the specific factors that contribute to community resilience and to develop interventions that foster these protective factors.

Interventions that address food security, mental health, and the psychological factors that promote adaptation to food-insecure environments are essential for promoting the well-being of individuals and communities in the face of climate change. Community-based approaches that empower individuals and communities to take action to address their own needs are particularly promising.

## Figures and Tables

**Table 1:** Description of model variables.

	*N*	T1				T2				*p*-Value
		Mean (%)	SD	95% CI		Mean (%)	SD	95% CI		
Food insecurity	362	13.6	7.3	12.8	14.3	11.4	6.5	10.7	12.1	<0.01
Anxiety	362	7.5	4.9	6.9	7.9	6.7	5.3	6.2	7.3	<0.01
Depression	362	8.1	7.1	7.3	8.8	6.4	6	5.8	7.1	<0.001
Average monthly income ($ = 130 KSH; May 2023)	362	34.5	35.9	—	—	—	—	—	—	—
Age (years)	362	41.7	12.6	—	—	—	—	—	—	—
Years of formal education	362	5.2	3.2	—	—	—	—	—	—	—
Married (1 = yes; 0 = no)	362	76%	0.43	—	—	—	—	—	—	—
Female gender	362	92%	0.27	—	—	—	—	—	—	—

*Note*: Mean (%), sd, and 95% confidence intervals are shown for primary variables at T1 and T2, and control variables at T1. *p*-values were calculated using the Wilcoxon sign-rank test for paired variables.

**Table 2:** Spearman rank correlation coefficients of all model variables.

	1	2	3	4	5	6	7	8
1. Food insecurity, T1	1	—	—	—	—	—	—	—
2. Food insecurity, T2	0.36*⁣*^*∗∗∗*^	1	—	—	—	—	—	—
3. Anxiety, T1	0.39*⁣*^*∗∗∗*^	0.22*⁣*^*∗∗∗*^	1	—	—	—	—	—
4. Anxiety, T2	0.24*⁣*^*∗∗∗*^	0.33*⁣*^*∗∗∗*^	0.29*⁣*^*∗∗∗*^	1	—	—	—	—
5. Depression, T1	0.26*⁣*^*∗∗∗*^	0.14	0.33*⁣*^*∗∗∗*^	0.15	1	—	—	—
6. Depression, T2	0.26*⁣*^*∗∗∗*^	0.27*⁣*^*∗∗∗*^	0.26*⁣*^*∗∗∗*^	0.49*⁣*^*∗∗∗*^	0.21*⁣*^*∗∗*^	1	—	—
7. Monthly income, T1	0.36*⁣*^*∗∗∗*^	−0.16	−0.12	0.01	−0.17	−0.01	1	—
8. Age (years), T1	0.02	0.02	−0.13	−0.14	0.09	0.01	−0.05	1
9. Years of formal schooling, T1	−0.28*⁣*^*∗∗∗*^	−0.36*⁣*^*∗∗∗*^	−0.05	−0.10	−0.09	−0.15	0.21*⁣*^*∗∗∗*^	−0.35*⁣*^*∗∗∗*^

*Note*: Spearman correlation coefficients shown of all primary (T1 and T2) and control (T1) variables. Bonferroni-adjustment applied to coefficient *p*-values.

*⁣* ^*∗*^ *p*  < 0.05.

*⁣* ^*∗∗*^ *p*  < 0.01.

*⁣* ^*∗∗∗*^ *p*  < 0.001.

## Data Availability

Study data are available upon reasonable request to the corresponding author.

## Supporting Information

Supporting Information Figure S1a, b presents food security conditions in the study area at the start and end of the study period, as reported by the Famine Early Warning Systems Network (FEWS NET). Table S1 provides the village-level averages (and standard deviations) for all primary study variables at both T1 and T2. These materials offer additional context to support interpretation of the main findings.
