# Supplementary material for: Navigating the Nexus of Food Insecurity, Anxiety, and Depression in the Face of Climate Change: A Longitudinal Study in Rural Kenya
Source: Depress Anxiety. 2025 Nov 7;2025:5510493. doi: 10.1155/da/5510493 (PMC12618132; doi:10.1155/da/5510493)
Supplement: Supporting Information — Figure S1a, b presents food security conditions in the study area at the start and end of the study period, as reported by the Famine Early Warning Systems Network (FEWS NET). Table S1 provides the village-level averages (and standard deviations) for all primary study variables at both T1 and T2. These materials offer additional context to support interpretation of the main findings. [file 5510493.f1.docx]

**Supplemental Figures 1a-b**:


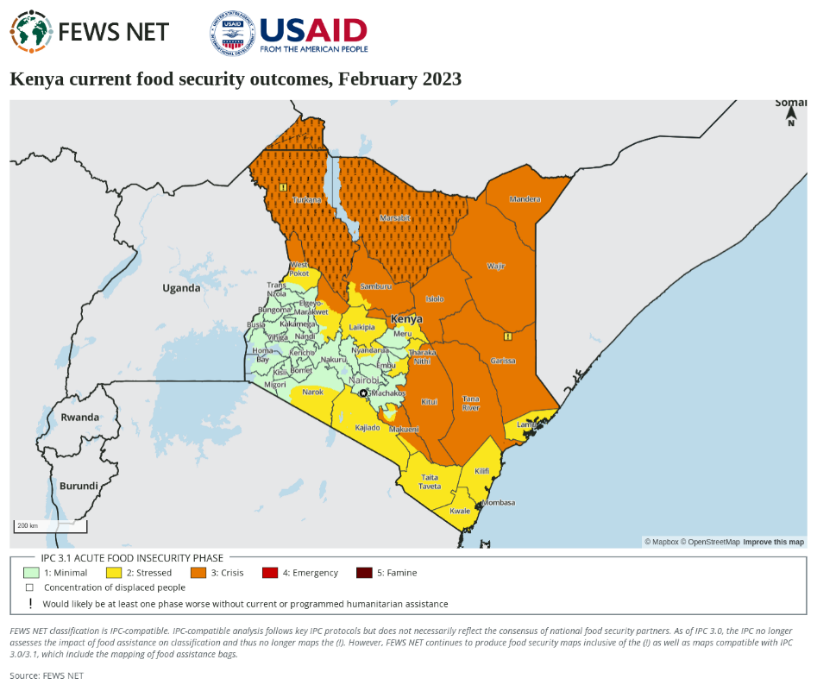


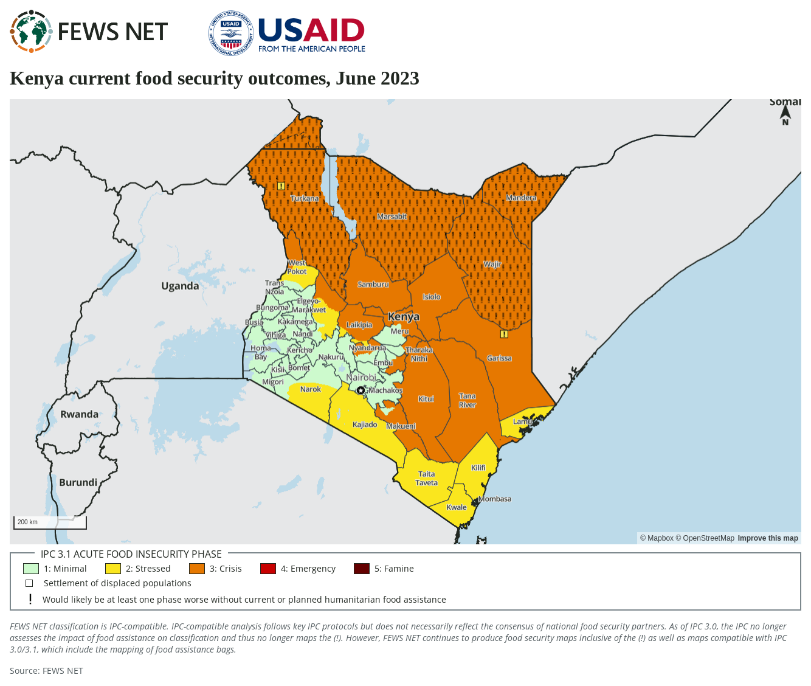


| Supplemental Table 1: Village-level aggregate key measures and distributions | | | | | | | | | | | | |
| --- | --- | --- | --- | --- | --- | --- | --- | --- | --- | --- | --- | --- |
|  | Depression, T1 | | Depression, T2 | | Anxiety, T1 | | Anxiety, T2 | | Food insecurity, T1 | | Food insecurity, T2 | |
| Village | Mean | SD | Mean | SD | Mean | SD | Mean | SD | Mean | SD | Mean | SD |
| 1 | 9.8 | 6 | 5 | 4.4 | 5.8 | 3.6 | 4.6 | 4.3 | 11 | 7.1 | 8.2 | 7.1 |
| 2 | 5.1 | 5.8 | 5.5 | 4.3 | 5.4 | 4.7 | 6 | 4.5 | 8.7 | 7.8 | 9.6 | 7.2 |
| 3 | 8 | 7.1 | 5.3 | 6.3 | 8.6 | 4.7 | 6.3 | 4.9 | 15.2 | 7 | 12 | 6.6 |
| 4 | 10.3 | 9.8 | 7.3 | 6 | 7.8 | 5.1 | 5.9 | 3.8 | 13.1 | 7.4 | 10.6 | 5.2 |
| 5 | 8.4 | 6.8 | 6.7 | 3.7 | 6.8 | 4.6 | 7.5 | 4.7 | 13.6 | 6.4 | 12.5 | 6.3 |
| 6 | 7.7 | 7.1 | 7.5 | 6.5 | 8.5 | 5.3 | 8 | 5.6 | 15.7 | 6.1 | 10.7 | 6.1 |
| 7 | 5.8 | 5.1 | 6.9 | 5.9 | 5.2 | 5.2 | 6.5 | 3.8 | 8.1 | 6.6 | 10.8 | 5.9 |
| 8 | 7 | 4.7 | 4.9 | 3.9 | 7.4 | 5.3 | 5.1 | 5.5 | 9.9 | 6.6 | 11.6 | 7.1 |
| 9 | 7.5 | 6.9 | 10.6 | 7.7 | 7.2 | 4.4 | 10.5 | 6.4 | 17.4 | 5.8 | 15.6 | 6.1 |
| 10 | 12.9 | 10 | 7.7 | 8 | 7.8 | 4.7 | 6.9 | 5.3 | 15 | 6.9 | 12.3 | 6.6 |
| Notes: Key measures averaged at the village-level with standard deviations | | | | | | | | | | | | |
